# A household randomized, controlled trial of the efficacy of 0.03% transfluthrin coils alone and in combination with long-lasting insecticidal nets on the incidence of *Plasmodium falciparum* and *Plasmodium vivax* malaria in Western Yunnan Province, China

**DOI:** 10.1186/1475-2875-13-208

**Published:** 2014-05-31

**Authors:** Nigel Hill, Hong Ning Zhou, Piyu Wang, Xiaofang Guo, Ilona Carneiro, Sarah J Moore

**Affiliations:** 1 London School of Hygiene and Tropical Medicine, Keppel St, London WC1E 7HT, UK; 2 Yunnan Institute of Parasitic Diseases, 6 Xiyuan Road, Simao, Puer, Yunnan, People’s Republic of China; 3 Environmental Health and Ecological Sciences Thematic Group, Ifakara Health Institute, Bagamoyo Research and Training Centre, Bagamoyo, Tanzania; 4 Swiss Tropical and Public Health Institute, Socinstr. 57, 4051 Basel, Switzerland; 5 University of Basel, Petersplatz 1, 4003 Basel, Switzerland

## Abstract

**Background:**

Mosquito coils are the most commonly used household insecticidal product in the world with sales exceeding 50 billion coils, used by two billion people worldwide annually. Despite strong evidence that coils prevent mosquito bites a systematic review concluded that there is no evidence that burning mosquito coils prevents malaria acquisition. Therefore, the current trial was designed to measure and compare prevention of malaria infection by mosquito coils or long-lasting insecticidal net (LLIN) or a combination of the two in Yunnan, China in the Greater Mekong sub-region.

**Methods:**

A four-arm single blind household-randomized design was chosen as coils emanate insecticide throughout the household. Households enrolled at baseline were randomly allocated by the lottery method to one of the four intervention arms: (i) nothing, (ii) 0.03% transfluthrin coils alone, (iii) deltamethrin long-lasting insecticide treated nets, (LLINs) alone or (iv) a combination of transfluthrin coils and deltamethrin LLINs. All household members were recruited to the study, with only those households excluded with pregnant or breastfeeding mothers, members with chest complaints or allergies or members that regularly slept away from home. The main outcome of interest was *Plasmodium falciparum* malaria prevalence detected by rapid diagnostic tests (RDTs) during six repeated monthly cross-sectional surveys. The secondary outcome of interest was the effect on *Plasmodium vivax* prevalence detected in the same way.

**Results:**

A total of 2,052 households were recruited into the study, comprising 7,341 individuals The odds ratios of testing positive by RDT with *P. falciparum* or *P. vivax* were >75% lower for all intervention arms compared with the control arm. Coils alone provided 77% protection (95% CI: 50%-89%), LLINs provided 91% protection (95% CI: 72%-97%) and the combination of coils and LLINs provided 94% protection (95% CI: 77%-99%) against *P. falciparum* compared with the control arm. There was no statistically significant difference between the protective efficacies of the different interventions.

**Conclusions:**

This is the first robust clinical evaluation of transfluthrin mosquito coils as a means to reduce malaria and the high degree of infection prevented would indicate they represent a potentially highly effective tool, which could be integrated into larger vector control programmes.

**Trial registration:**

ClinicalTrials.gov Identifier: NCT00442442, March 2007.

## Background

Mosquito coils are the most commonly used household insecticidal product in the world with sales exceeding 45 to 50 billion coils used by 2 billion people worldwide each year [1]. The popularity of coils is due to their low cost at $0.02 each [2], their ability to be used without electricity or equipment and cultural acceptance, because smoke is used in many cultures to drive away mosquitoes [3]. These products present a great opportunity for public health, because such products could provide a means of disease control that is already proven highly acceptable to end-users and has undergone stringent safety testing.

Despite strong evidence that coils prevent mosquito bites, a systematic review concluded that there is no evidence that burning mosquito coils prevents malaria acquisition [4,5]. Therefore, the current trial was designed to measure and compare prevention of malaria infection by mosquito coils or long lasting insecticidal net (LLIN) or a combination of the two. The trial was undertaken in Yunnan, a region of China in the Greater Mekong sub-region where malaria remains endemic despite substantial investment in LLIN programmes [6] and artemisinin resistant malaria threatens worldwide initiatives to control and eliminate malaria [7]. The product selected for evaluation was a 0.03% transfluthrin mosquito coil (RAID®, SC Johnson, USA). Transfluthrin is a highly effective fast-acting pyrethroid insecticide used extensively in household and hygiene products, mainly against flying insects, such as mosquitoes and flies. The WHO have carried out an evaluation of the extensive toxicity literature available on transfluthrin and concluded that transfluthrin is: “unlikely to present acute hazard in normal use” [8].

Centrally organized vector-based malaria control programmes have never incorporated the use of coils as a methodology, presumably as there is a lack of clinical evidence of efficacy, yet there is little doubt over their effects on preventing mosquitoes from biting [9]. A large proportion of global malaria transmission occurs before sleeping hours and outdoors due to outdoor and early biting malaria vectors [10], which are of particular importance in Southeast Asia. Given that mosquito coils are known to be effective at reducing biting, they are relatively cheap, universally available, purchased and used by so many households, there is clearly a need to examine more closely their potential for use in integrated vector control programmes. The study tested the hypothesis that using transfluthrin mosquito coils, LLINs or a combination of both interventions would reduce 1) the incidence of *Plasmodium vivax* and *Plasmodium falciparum* malaria and 2) numbers of malaria vector mosquitoes attempting to feed on humans, among those households using the interventions relative to those households not using any of the interventions other than their normal malaria prevention practices.

## Methods

### Study design

A household-randomised design was chosen as coils emanate insecticide throughout the household, and LLINs are also know to have a protective effect at the household level. Field workers and participants were not blinded to treatment allocation, as this was impossible in practice. However, the field staff collecting monthly RDT data were not aware of the intervention which individuals had been using thus achieving single blinding (investigator) of the study. Furthermore, microscopists at Yunnan Institute of Parasitic diseases that verified positive RDTs by miscoscopy and the statistician was blind to the allocation. The intervention of interest was the use of 0.03% transfluthrin coils (SC Johnson), deltamethrin long-lasting insecticide-treated nets (LLINs) (TianJin-Yorkool Ltd, Tianjian, PR China, and Lantrade Global Supplies Ltd, Gerrards Cross, UK), or a combination of transfluthrin coils and deltamethrin LLINs compared with a non-intervention control arm. The study was designed to investigate the benefits of using mosquito coils with or without LLINs on malaria prevention. The main outcome of interest was *P. falciparum* malaria prevalence detected by rapid diagnostic tests (RDTs) during six repeated monthly cross-sectional surveys. The secondary outcome of interest was the effect on *P. vivax* prevalence detected in the same way.

### Participants

The study was conducted in Ruili County, Yunnan Province, P.R. China, close to the Myanmar border between April and October 2007. Yunnan is one of only two provinces in China that remains malaria endemic and Ruili County has a high number of cases due to proximity of heavily forested areas, a high proportion of migrant populations moving over the border between countries and the remote location of minority group habitations which are difficult to cover with centralized vector control and public health programmes. All communities enrolled were in rural areas. The border area stretches 170 km with a population of 111,449 people. The average elevation was 780 metres with mean temperature of 20°C and annual average rainfall 1,394.8 mm. During 2005 to 2006, 1,125 malaria cases were recorded, of which 31% was *P. falciparum*[11]. The main malaria vectors are *Anopheles minimus* and *Anopheles sinensis*.

Villages selected were Leiying, Dengga, Leilong, Huyiu, Banling, Hulan, Mendian, Najingli, Ruili farm, due to their relatively high annual malaria incidence. The total population is 4832 households, with 789 malaria cases (approximate annual malaria incidence of 0.02) with 254 cases of *P. falciparum* (32%) during 2005 [11]. Prior to the clinical study there has never been any organized vector control in the area but residents burn fires and mosquito coils and some use untreated bednets for protection against bites [12].

### Recruitment and allocation

The recruitment process began with a meeting between the village head/elders and the local malaria control staff who explained the study to the villagers. As there is a possibility that mosquitoes could be repelled from entering a house using coils or an LLIN and thus being actively diverted to a neighbouring house nearby only those households that were more than 20 m apart were enrolled, and no two adjacent houses were used. In addition, to ensure potential diversion was kept to an absolute minimum and did not lead to bias a maximum of 20% of the houses in any village were recruited into the study. In this way only 15% of houses in any village had a study intervention, so any diverted mosquitoes would not be measured in study households, but would be absorbed by the large number of houses not participating in the study.

Given the protocol inclusion/exclusion criteria of a maximum of 20% of households in any village being available for the study (to negate diversion bias), the number of households/village was calculated. From this list, those villages that had reported malaria cases in the previous 48 months were selected. Once houses eligible and happy to participate in the study were identified the study coordinator randomly assigned them to treatment groups by drawing lots. Members of the field team then recruited all household inhabitants to the study on written informed consent. Households were excluded if household members included 1) children under six years, 2) pregnant or breastfeeding mothers, 3) those with chest complaints or allergies, and 4) those that regularly slept away from home. This was done to ensure that participants were healthy and not likely to contract malaria outside of the household. However, in practice children under six years of age were included in the study with their parent or guardian’s consent.

### Intervention

Those people allocated to LLIN were provided with an new LLIN free of charge for each sleeping space, those allocated to coils were given two coils to burn each evening, those allocated to LLIN + coils were given both products and the untreated control group continued to use their own personal protection methods. It would be unethical to ask anyone not to do this but a record was kept of such ad-hoc coil use in the negative control group and those reporting the use of one box or more (10 coils/5 nights) were excluded from the analysis for that round. At each monthly visit, the households in the coil and the coil+LLIN groups were given sufficient coils to last for the next month, and the empty boxes of the previous month were collected as a compliance check.

Households were permitted to use up to two coils/night as it is common practice for the family to sit in a living area in the early evening and then retire to a separate bedroom area at night. Where houses had separate living and sleeping areas it was thus possible to light one coil early in the evening in the living area, then light another in the sleeping area before the family retired to bed, overcoming problems of moving coils once alight.

### Compliance

Every night in the village, the village leader was requested to check whether his village used the coils or nets; and every month a questionnaire was delivered on compliance to household heads enrolled in the study. Individuals were asked about the number of night’s use of the intervention to which they had been allocated (coils, LLINs or both), or use of interventions to which they had not been allocated (coil-use in the control group). There were no LLINs in the control arm. As a further check of compliance the empty boxes of the previous month were collected to confirm they had been used.

### Data collection

A baseline survey was undertaken among household heads, along with the first cross-sectional malaria survey after randomization in April 2007 to collect data on socio-demographic factors including age, sex, level of education, occupation and history of malaria in the previous 12 months. Presence of malaria infection among all household members was detected using CareStart pLDH Malaria G0121 (AccessBio Inc., Monmouth, USA) rapid diagnostic tests that distinguished *P. falciparum* and *P. vivax* parasites during six cross-sectional surveys carried-out monthly between May and October 2007. For those RDTs that showed a positive result a thick film blood slide was taken for verification by Microscopy at Yunnan Institute of Parasitic Diseases. Data were also collected on the reported history of fever in the previous month and use of treatment including anti-malarial drugs. All positive cases were referred to the local health centre to receive appropriate anti-malaria treatment. Treatment for *P. vivax* was chloroquine (1.5 g) + primaquine (180 mg), while treatment for *P. falciparum* was artesunate (600 mg) + primaquine (45 mg). The protocol stated that patients should be excluded if they contracted malaria to prevent bias from household clustering of malaria infections. However, on analysis, data showed that there was a protocol deviation and no patients that contracted malaria were excluded in practice. All malaria cases were treated with primaquine, which clears hypnozoites from the liver, excluding the possibility of recrudescence biasing data from the trial on malaria incidence.

### Entomology

As an additional outcome measure a series of entomological monitoring studies were included within the main clinical trial. One of the main villages, Dennggar, included in the study was chosen as a sentinel site where entomological collections were made over a 4-month period mid study (July – October). Standard CDC light traps (Bioquip Inc., USA) were hung indoors close to an occupied bed each night in four houses from each of the four treatment groups (n = 16). Traps were set in the evening before dusk and left running all night. In the morning, all mosquitoes were collected from each trap, knocked down by chilling, then identified by local experts. The number of mosquitoes indoors was recorded in each study group to determine whether any treatment was significantly reducing mosquito entry into houses. Data were analysed as a proportion of mosquitoes, per house, per night. It should be noted that for the control arm, houses with untreated bednets were selected as the controls to ensure that the CDC light trap catch was optimised and could be compared to the protective efficacy of each the interventions to the user sleeping next to the light trap.

### Sample size

A sample size of 400 households per intervention arm, each with an average of five individuals, was calculated to detect a 50% reduction in *P. falciparum* incidence in the households using transfluthrin coils compared with those not allocated to an intervention with 90% power and 95% significance [13]. A total of 2,052 households were recruited into the study at baseline (target number was 1,600) comprising 7,341 individuals (target number was 8,000). Households enrolled at baseline were randomly allocated by the lottery method to one of the four intervention arms (i) nothing, (ii) coils alone, (iii) LLINs alone or (iv) coils and LLINs. As the study was clustered at the household level the increase in numbers of households recruited was a positive occurrence. The slightly lower total number of individuals was presumably due the smaller family size per house of four rather than five, as used in the sample size estimate.

As there is a possibility that mosquitoes could be repelled from entering a house using coils or an LLIN and thus being actively diverted to a neighbouring house nearby only those households that were more than 20 m apart were enrolled, and no two adjacent houses were used. In addition, to ensure potential diversion was kept to an absolute minimum and did not lead to bias we only recruited a maximum of 20% of the houses in any village into the study. In this way only 15% of houses in any village had a study intervention, so any diverted mosquitoes would not be measured in study households, but would be absorbed by the large number of houses not participating in the study.

### Data analyses

The data were analysed using Stata 11.0 (StataCorp. 2009. Stata Statistical Software: Release 11. College Station, TX: StataCorp LP.). Baseline socio-demographic variables (age, sex, education level of adults) were compared between the intervention arms. The main analysis was an intention-to-treat analysis according to the groups to which individuals had been randomised. No stratification by village was used. A secondary per-protocol analysis restricted data to those with > 90% reported compliance for the month prior to each cross-sectional survey, equivalent to non-compliance of three days or less per month. A random-effects logistic regression model adjusting for repeated sampling of the same individual was used to identify potential confounding factors such as age group and level of education among adults in the control group. A multilevel, mixed-effects logistic regression model was used to estimate odds ratios (OR) of each intervention (fixed effect) compared with the control arm, using nested random effects to adjust for the non-independence of observations from individuals in the same household, and for repeated sampling of the same individual. The protective efficacy was estimated as (1 – OR) × 100%.

### Ethics

LSHTM University of London Ethics Committee and the Yunnan Bureau of Health approved the study. At the end of the study, all households that had not been provided with an LLIN (the coil only group and the negative control group) were given LLINs as required by the ethics committee to ensure parity among all participants.

## Results

### Baseline data

**Figure 1:**
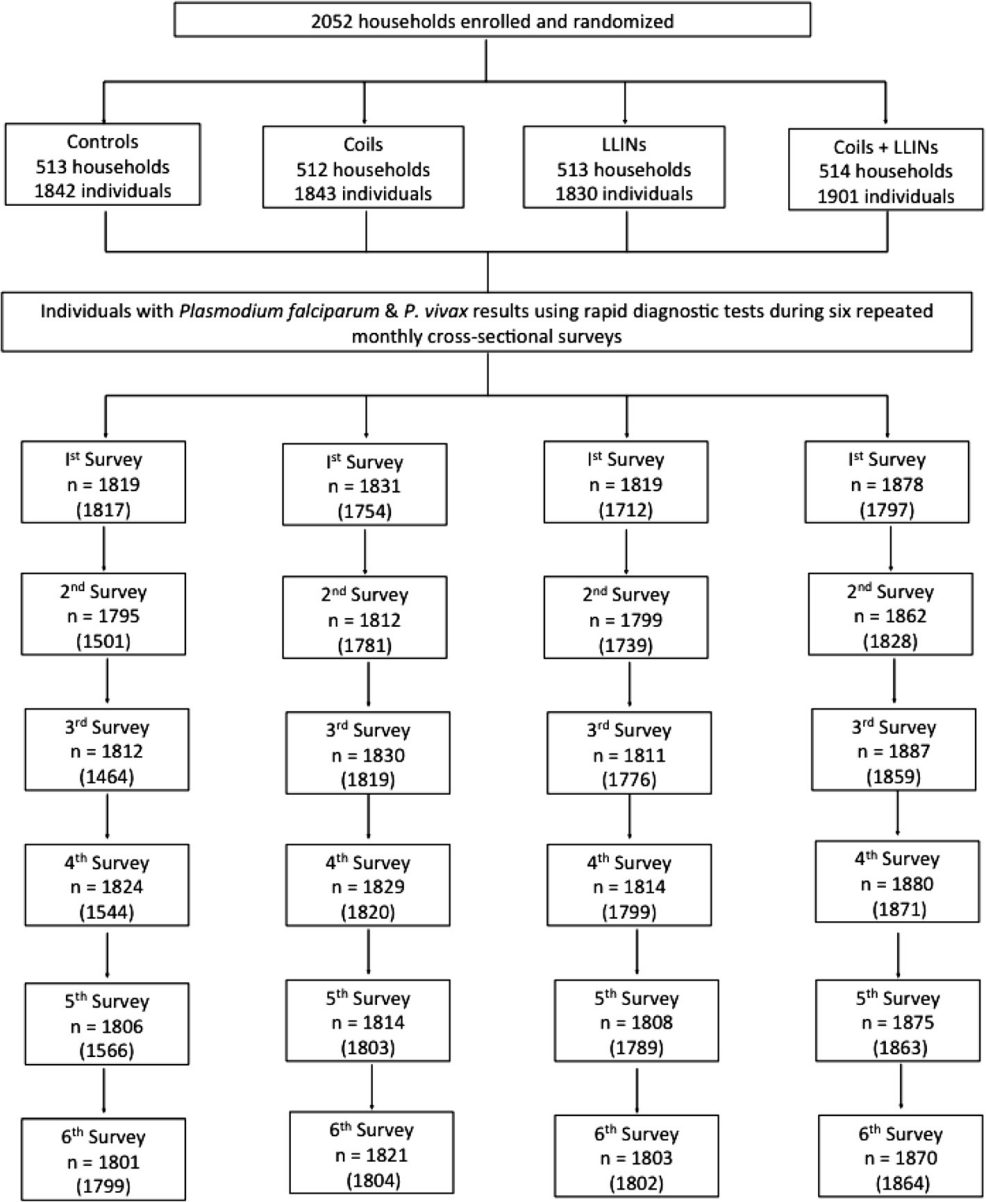
**Study flowchart.** Numbers in parentheses are for per-protocol analysis i.e. >90% compliance in previous month with intervention allocated.

Over 75% of the study participants were over 16 years of age, 75% of these had primary level education or above and the main occupation of the head of household was as a farmer (83%). Age, gender, level of education among adults and reported history of malaria in the previous 12 months was evenly distributed between the intervention arms (Table 1). Reported history of malaria in all the arms corresponded to that at County level (0.02 – 0.03 cases per year). Loss to follow up was less than 2% in each of the four treatment arms, with lowest loss to follow up in the LLIN only arm (1.5%), loss to follow up of 2% in the coil only arm and 1.9% in the coil & LLIN arm although these were not significantly different (Figure 1).

**Table 1:** Baseline socio-demographic characteristics of individuals enrolled by intervention arm

	**Control**	**Coils**	**LLINs**	**Coils + LLINs**
Individuals enrolled	1841	1843	1828	1901
Female (%)^§^	919 (49.9)	911 (49.4)	918 (50.2)	924 (48.6)
Mean age (years) [s.d.]	34.2 [17.9]	33.9 [18.2]	34.3 [17.7]	34.5 [18.2]
≥15 years old (%)^§^	1464 (79.7)	1428 (77.5)	1451 (79.3)	1498 (78.9)
Level of education if ≥15 years old (%)^§^	n = 1456	n = 1425	n = 1434	n = 1497
None/limited	384 (26.4)	338 (23.7)	358 (25.0)	378 (25.2)
Primary	444 (30.5)	471 (33.1)	424 (29.6)	495 (33.1)
Secondary	628 (43.1)	616 (43.2)	652 (45.5)	624 (41.7)
History of malaria in previous 12 months (%)^§^	61 (3.3)	43 (2.4)	43 (2.4)	63 (3.3)

^§^Discrepancies in percentages are due to missing data for some characteristics.

### Malaria infection and potential confounders

The prevalence of *P. falciparum* infection was generally low, ranging from 0.11 to 0.66% in the control arm, while *P. vivax* infection prevalence varied from 0 to 0.72% in the control arm (Table 2). There were no mixed species infections. No individuals were positive for *P. falciparum* more than once, while four individuals tested positive for *P. vivax* twice and one individual tested positive three times.

**Table 2:** Number (%) positive over results available by rapid diagnostic test from of six repeated monthly cross-sectional surveys

	**Control**		**Coils**		**LLINs**		**Coils + LLINs**	
	***P. falciparum***	***P. vivax***	***P. falciparum***	***P. vivax***	***P. falciparum***	***P. vivax***	***P. falciparum***	***P. vivax***
Survey 1	5/1819 (0.27)	0/1819 (0)	1/1831 (0.05)	0/1831 (0)	1/1819 (0.05)	0/1819 (0)	1/1878 (0.05)	0/1878 (0)
Survey 2	3/1795 (0.17)	4/1795 (0.22)	2/1812 (0.11)	1/1812 (0.06)	0/1799 (0)	3/1799 (0.17)	0/1862 (0)	1/1862 (0.05)
Survey 3	6/1812 (0.33)	13/1812 (0.72)	0/1830 (0)	2/1830 (0.11)	1/1811 (0.06)	0/1811 (0)	0/1887 (0)	0/1887 (0)
Survey 4	12/1824 (0.66)	3/1824 (0.16)	3/1829 (0.16)	4/1829 (0.22)	1/1814 (0.06)	2/1814 (0.11)	0/1880 (0)	1/1880 (0.05)
Survey 5	7/1806 (0.39)	9/1806 (0.50)	1/1814 (0.06)	1/1814 (0.06)	0/1808 (0)	3/1808 (0.17)	1/1875 (0.05)	1/1875 (0.05)
Survey 6	2/1801 (0.11)	9/1801 (0.50)	1/1821 (0.05)	0/1821 (0)	0/1803 (0)	1/1803 (0.6)	0/1870 (0)	0/1870 (0)

An investigation of potential risk factors for malaria in the control group identified a weak association between age and *P. falciparum* infection with those aged ≥15 years having an odds ratio of 4.31 compared with those aged 2–14 years (95% CI: 0.93, 19.98; p = 0.062). There was also some weak evidence of an association between level of education among adults and *P. falciparum* infection in the control group. Those with primary education had an odds ratio of 0.40 compared with those with no or limited education (95% CI: 0.15, 1.04; p = 0.061), but this effect was not significant for those with secondary or higher education (p = 0.148). Neither covariable showed a significant association with *P. vivax* prevalence in the control arm.

### Efficacy of the intervention

The unadjusted odds ratios of testing positive by RDT with *P. falciparum* or *P. vivax* were considerably lower for all intervention arms compared with the control arm (see Table 3). The confidence intervals overlapped between all arms, suggesting no significant difference between the protective efficacies of the different interventions, although the magnitude of effect was greatest for the combined intervention of LLINs plus coils. As there were no repeat positives or evidence of household clustering for *P. falciparum*, the mixed-effects, multi-level model gave almost identical results to a random effects model adjusting only for repeated observations of the same individual. Age and level of education among adults (≥15 years old) were tested separately for inclusion in a multivariable random effects model, and education was no longer significant (p > 0.1). Adults were nearly five times more likely to be infected with *P. falciparum* than children, with an odds ratio of 4.86 (95% CI: 1.18, 20.05; p = 0.029). The odds ratios for the interventions, adjusted for the effect of age, were almost identical to the unadjusted odds ratios (Table 3). As there was no evidence of an association of either age or education with *P. vivax* infection among controls, these factors was not tested for inclusion in a multivariable model of *P. vivax* infection.

**Table 3:** Odds ratios and protective efficacy of interventions using an intention to treat analysis, adjusting for household clustering and repeated observation of individuals with mixed-effects logistic regression

	**Control**	**Coils**	**LLINs**	**Coils + LLINs**
Odds Ratio of being P. falciparum positive	1	0.23	0.09	0.05
(95% Confidence Interval [CI])	-	(0.10,0.49)	(0.03, 0.28)	(0.01, 0.23)
Age-adjusted OR	-	0.23	0.09	0.06
(95% CI)	-	(0.11, 0.50)	(0.03, 0.28)	(0.01, 0.23)
p-value§		0.0002	<0.0001	<0.0001
Protective efficacy		77%	91%	94%
(95% CI)		(50, 89)	(72, 97)	(77, 99)
Odds Ratio of being P. vivax positive	1	0.20	0.21	0.07
(95% Confidence Interval [CI])	-	(0.09, 0.44)	(0.10, 0.47)	(0.02, 0.24)
p-value	-	<0.0001	0.0001	<0.0001
Protective efficacy		80%	79%	93%
(95% CI)		(56, 91)	(53, 90)	(76, 98)

^§^P-values for unadjusted and age-adjusted odds ratios were identical.

The level of compliance with the allocated interventions was high: > 94% of individuals used the coils and/or LLINs for > 90% of the month prior to the surveys. Conversely, those in the control arm were less likely to follow the request of the study directors to not use any intervention, with 13-19% using local coils for 3 or more days in the month prior to the survey. A per-protocol analysis including only those with > 90% compliance gave almost identical results to the intention-to-treat analysis.

### Entomology

Mosquito densities were lower in all treatment groups with a >80% reduction from the use of LLINs or mosquito coils compared to an untreated bed net (Table 4).

**Table 4:** Arithmetic mean monthly indoor mosquito catch/house/night

	**Control**	**LLIN**	**Coils**	**Coils + LLIN**
**July**	22	6	5	3
**August**	38	8	2	4
**September**	15	1	2	0
**October**	12	1	1	0
**Mean**	21.75	4	2.5	1.75
**Std. Dev.**	11.6	3.6	1.7	2.1
**% reduction**	-	82%	88%	92%

## Discussion

Compared to the untreated control arm, both *P. falciparum* and *P. vivax* malaria cases in the group using mosquito coils were reduced by more than 75%, a remarkably high reduction in infection. The level of malaria prevention afforded by coils was very similar to that of the LLIN in the same study. When the two methods were employed in combination, the reduction in malaria infection was increased further to more than 90%. This may indicate that malaria in this area is likely to be transmitted by more than one vector with a mixture of biting behaviour over a prolonged period of the evening and night. Indeed, entomological data collected from the area at the same time as the trial (July 2007) indicated that *An. sinensis* was the most abundant malaria vector comprising 50% of Anophelines collected, in addition to smaller numbers of *Anopheles kochi, Anopheles splendidus, Anopheles barbirostris, Anopheles vagus, Anopheles jeyporiensis, Anopheles annularis, Anopheles philippinsis, Anopheles minimus, Anopheles tessallatus, Anopheles maculatus, Anopheles barbumbrosus, Anopheles dirus* and *Anopheles culicifacies*[12]. One might speculate that the coils are having a greater effect in the evening before people retire to bed, and that the LLIN is further preventing infection later into the night, but this would need additional investigation.

Given that coils are universally sold and used in most malaria risk areas globally, they are readily accepted by the local population (as they reduce nuisance biting) and are relatively inexpensive at around 2 cents per coil, it would seem that the high reduction in infections demonstrated here could make transfluthrin mosquito coils suitable for inclusion in some vector-borne disease control programmes.

As only a single type of coil (Raid®) was evaluated, which is known to be of high quality, the results obtained should not automatically be extrapolated to other products available in the market. Similarly, the study was conducted in rural areas of S.E. Asia and other locations are likely to have different habitats and vector species [14], which are likely to influence the success of coils to some degree. The current study investigated entomological and malaria infection outcomes and monitored use and acceptance of the various interventions. However, there was no attempt to measure clinical malaria or to record other physical or physiological effects of either the LLIN or the smoke from the coils [1] and these aspects may need to be taken into consideration if coil use were to be promoted more widely.

## Conclusions

This is the first robust clinical evaluation of coils as a means to reduce malaria and the high reduction in infections achieved would indicate that they represent a potentially highly effective tool, which could be implemented at the household or community level, or integrated into larger vector control programmes.

## Competing interests

The authors declare that they have no competing interests. SC Johnson had no role in study design, data collection or data analysis.

## Authors’ contributions

NH devised the study, was responsible for the protocol development, obtained funding. HNZ assisted in development of the protocol, acted as study coordinator in China, trained local field staff, initiated the practical phase of the project, and assisted in production of the manuscript. PW and XG acted as main investigators in the field sites. IC assisted in the study design, undertook statistical analysis of results, and contributed to the manuscript. SJM wrote the manuscript. All authors read and approved the final version of the manuscript.
